# Transcriptome Analysis Reveals Comprehensive Insights into the Early Immune Response of Large Yellow Croaker (*Larimichthys crocea*) Induced by Trivalent Bacterial Vaccine

**DOI:** 10.1371/journal.pone.0170958

**Published:** 2017-01-30

**Authors:** Xin Zhang, Yinnan Mu, Pengfei Mu, Jingqun Ao, Xinhua Chen

**Affiliations:** 1 School of Marine Sciences, Ningbo University, Ningbo, China; 2 Key Laboratory of Marine Biogenetic Resources, Third Institute of Oceanography, State Oceanic Administration, Xiamen, China; 3 Fujian Collaborative Innovation Center for Exploitation and Utilization of Marine Biological Resources, Xiamen, China; 4 Laboratory for Marine Biology and Biotechnology, Qingdao National Laboratory for Marine Science and Technology, Qingdao, China; National Cheng Kung University, TAIWAN

## Abstract

Vaccination is an effective and safe strategy for combating bacterial diseases in fish, but the mechanisms underlying the early immune response after vaccination remain to be elucidated. In the present study, we used RNA-seq technology to perform transcriptome analysis of spleens from large yellow croaker (*Larimichthys crocea*) induced by inactivated trivalent bacterial vaccine (*Vibrio parahaemolyticus*, *Vibrio alginolyticus* and *Aeromonas hydrophila*). A total of 2,789 or 1,511 differentially expressed genes (DEGs) were obtained at 24 or 72 h after vaccination, including 1,132 or 842 remarkably up-regulated genes and 1,657 or 669 remarkably down-regulated genes, respectively. Gene ontology and Kyoto Encyclopedia of Genes and Genomes enrichments revealed that numerous DEGs belong to immune-relevant genes, involved in many immune-relevant pathways. Most of the strongly up-regulated DEGs are innate defense molecules, such as antimicrobial peptides, complement components, lectins, and transferrins. Trivalent bacterial vaccine affected the expressions of many components associated with bacterial ligand–depending Toll-like receptor signaling pathways and inflammasome formation, indicating that multiple innate immune processes were activated at the early period of vaccination in large yellow croaker. Moreover, the expression levels of genes involved in antigen processing were also up-regulated by bacterial vaccine. However, the expression levels of several T cell receptors and related CD molecules and signal transducers were down-regulated, suggesting that the T cell receptor signaling pathway was rapidly suppressed after vaccination. These results provide the comprehensive insights into the early immune response of large yellow croaker to vaccination and valuable information for developing a highly immunogenic vaccine against bacterial infection in teleosts.

## Introduction

Large yellow croaker (*Larimichthys crocea*) is one of the most important aquaculture species in China, with the annual yield exceeding any other net-cage-farmed marine fish species [1]. However, the diseases caused by bacteria, such as *Vibrio parahaemolyticus* [2], *Vibrio alginolyticus* [3], and *Aeromonas hydrophila* [4], outbreak frequently and result in tremendous economic losses in large yellow croaker aquaculture industry. Chemotherapeutants and antibiotics are useful for preventing bacterial infection, but long term treatment often leads to resistance and environmental pollution [5]. Vaccination is an effective and safe strategy for combating bacterial diseases in fish [6]. A number of vaccines are commercially available for use in the aquaculture industry, such as formalin-inactivated *Yersinia ruckeri* [7], *Aeromonas salmonicida* [8], and *Vibrio anguillarum* [9]. The effects of vaccines were mainly evaluated by serum or mucus antibody titers and survival rate [10]. Recently, several studies on the gene expression changes have been performed to understand the immune response to bacterial vaccines in fish. In Atlantic salmon (*Salmo salar*), the increased expressions of antibacterial proteins and proteases were observed after vaccination with live *Aeromonas salmonicida* [11]. In zebrafish (*Danio rerio*), multiple pathways including acute phase response, complement activation, and antigen processing and presentation were remarkably affected during the early stage of vaccination [12]. The genes involved in inflammation and antioxidant defense were also up-regulated in Atlantic cod (*Gadus morhua*) following vaccination with heat-killed *Vibrio anguillarum* [13].

An inactivated trivalent bacterial vaccine, consisting of *V*. *alginolyticus*, *V*. *parahaemolyticus*, and *A*. *hydrophila*, was developed and provided an effective protection against bacterial infection in large yellow croaker, with a relative percent survival (RPS) of 88.9% [14]. This trivalent bacterial vaccine has been used to study the expression patterns of immune-relevant genes in large yellow croaker, such as goose-type lysozyme [15], caspase 9 [16], C-type lectin-like receptor [17], CXCL8 [18], CXCL13 [19], and stefin [20], indicating that this bacterial vaccine could induce the expression of innate immunity molecules in large yellow croaker. Currently, high-throughput RNA sequencing (RNA-seq) technology provides a powerful and cost-effective approach to understand the immune response evoked by different stimulus. For instance, RNA-seq was applied to identify immune-candidate genes, infection markers, and putative signaling pathways in rainbow trout (*Oncorhynchus mykiss*) [21], tilapia (*Oreochromis niloticus*) [22], and grass carp (*Ctenopharyngodon idellus*) [23] after bacterial infection.

Here, we used RNA-seq to examine the transcriptional profiles of the large yellow croaker spleens at early time points (0, 24, and 72 h) following immunization with inactivated trivalent vaccine (*V*. *alginolyticus*, *V*. *parahaemolyticus* and *A*. *hydrophila*). A series of differentially expressed genes (DEGs) were identified and found to be involved in the innate immunity and acquired immunity processes. Moreover, quantitative real-time PCR was used to verify expression changes of partial immune-relevant genes. These data provide valuable information to better understand the early immune response after vaccination in large yellow croaker.

## Materials and Methods

### Ethics statement

All handling of fish was approved by the Institutional Animal Care and Use Committee of Fujian Province, and performed in strict accordance with the Regulations for the Administration of Affairs Concerning Experimental Animals, under protocol license number: SYXK (MIN) 2007–0004. All surgeries were carried out under Tricaine-S anesthesia, and all efforts were made to minimize suffering.

### Inactivated trivalent bacterial vaccine and fish samples

The *V*. *parahaemolyticus*, *V*. *alginolyticus* and *A*. *hydrophila* were isolated from the diseased large yellow croaker previously [14]. For vaccine preparation, the three bacteria were cultured in Luria-Bertain (LB) for 16 h in a shaker incubator (180 rpm) at 30°C, respectively. After collected and washed with the sterilized phosphate-buffered saline (PBS, pH 7.4), *V*. *parahaemolyticus*, *V*. *alginolyticus* and *A*. *hydrophila* were mixed at same proportion (1.0×10^9^ CFU/mL each) and inactivated with 0.5% formalin (v/v) at 4°C for 4 h. The mixed inactivated trivalent vaccine was washed with the sterilized PBS. To evaluate the effectiveness of the formalin-inactivation, 50 μL of formalin-inactivated suspensions were spread on LB agar (1.5%, w/v) and incubated at 37°C for 24 h. No bacterial growth was observed, indicating that the bacterial cells were completely inactivated.

Large yellow croakers (length: 20 ± 1.76 cm; weight: 100 ± 21.5 g) were obtained from the Mari-culture farm in Lianjiang, Fuzhou, China. The fish were maintained at 20°C in aerated water tanks with a flow-through seawater supply. After one week of acclimatization, a group of 30 fish were injected intraperitoneally with inactivated trivalent bacterial vaccine (1.0×10^9^ CFU/mL each) at a dose of 0.2 mL/100 g fish. The spleen tissues were harvested from 10 large yellow croakers at each time point (0, 24, and 72 h) after vaccination.

### RNA extraction, library preparation, and sequencing

Total RNA was isolated from pooled spleen tissues of 6 fish sampled at each time point for RNA-seq analysis using TRIZOL® Reagent (Invitrogen, USA), respectively, and treated with RNase-free DNase I (Takara, China) at 37°C for 1 h to remove residual genomic DNA. The RNA samples (5 μg each) were used to construct RNA-seq libraries by Illumina mRNA-Seq Prep Kit, and the libraries were sequenced by paired-end sequencing on the Illumina HiSeq 2000 sequencing platform (Illumina, USA).

### Identification of the differentially expressed genes

Clean reads were picked out from the raw reads of each library following removal of the reads containing adaptor sequences, reads with an N (unknown bases in read) percentage higher than 10%, and low quality reads (> 50% of bases with a quantity score Q-value ≤ 10) using an in-house C^++^ program. The clean reads were aligned to the large yellow croaker genome (**JRPU00000000**) [24] using the software Burrows-Wheeler Aligner (BWA, parameter: -o 1 -e 63 -i 90 -L -k 2 -l 31 -t 4 -q 10) [25], and mapped to the coding sequences (CDS) of large yellow croaker genome using Bowtie2 (Parameter: -q --phred64 --sensitive --dpad 0 --gbar 99999999 --mp 1,1 --np 1 --score-min L,0,-0.1 -I 1 -X 1000 --no-mixed --no-discordant -p 1 -k 200) [26]. The gene expression levels were calculated based on FPKM (fragments per kilobase of transcripts per million fragments mapped) values by using RSEM (RNA Seq by Expectation Maximization) with default setting [27]. Comparisons of gene expression difference between control (0 h) and other time points after vaccination (24 and 72 h) were performed by strict Poisson distribution algorithm. Genes with the absolute value of the log_2_Ratio ≥ 1 and FDR (false discovery rate) ≤ 0.001 were defined as DEGs [28].

### Gene ontology and kyoto encyclopedia of genes and genomes (KEGG) analysis

All the DEGs were mapped to the gene ontology (GO) database (http://www.geneontology.org/) using Blast2GO software [29]. KEGG Orthology (KO) was carried out by BLASTx based on the KEGG database (http://www.genome.jp/kegg/). Finally, GO and KO enrichment analysis were performed according to the hypergeometric distribution test. For GO enrichment analysis, all of the *P*-values were performed with Bonferroni correction. We have selected a corrected *P*-value of 0.05 as a threshold to determine significant enrichment of the gene sets. In contrast, for KO enrichment analysis, we have used a Q-value of 0.05 as the threshold to determine significant enrichment of the gene sets.

### Quantitative real-time PCR

Quantitative real-time PCR was performed using Mastercycler epgradient realplex4 (Eppendorf, Germany) with SYBR Green (Takara, China). Primer sets were designed based on the coding sequences of identified genes in large yellow croaker genome and specific PCR product of each gene was observed on the agarose gels (**S1 Table** and **S1 Fig**). Total RNA was extracted from pooled spleen tissues of three fish sampled at each time point (0, 24, and 72 h) after vaccination for Real-time PCR analysis. Real-time PCR was performed in a total volume of 20 μL, and cycling conditions were 95°C for 1 min, followed by 40 cycles of 95°C for 15 s, 58°C for 15 s, 72°C for 20 s. Each real-time PCR assay was repeated three times with different batches of fish. The expression level of target gene was normalized by that of β-actin using the 2^-ΔΔCT^ method [30]. The fold change was calculated as the average expression level of target gene in trivalent vaccine-challenged samples at each time point (24 and 72 h) divided by that in the control sample (0 h). The data of real-time PCR was analyzed using GraphPad Prism 5 software and expressed as the standard error of the mean (SEM). Two-tailed Student’s *t* test was used for the significance test between the experimental group and the control group. A *P*-value <0.05 was considered to be statistically significant.

## Results

### Sequence analysis of the transcriptomes

Three transcriptomes were sequenced from pooled spleens of large yellow croaker after vaccination. More than 5.4 gigabase (Gb) of data were generated in each library (**Table 1**). After eliminating adaptor sequences and low-quality sequences, a total of 63,568,408, 60,346,074, and 60,445,278 clean reads were obtained from the three libraries (0, 24, and 72 h), respectively. Average 70% of the clean reads could be mapped to the genome of large yellow croaker with 19,648, 19,106, and 19,145 genes, respectively. Raw sequencing reads data has been deposited in SRA (NCBI) under the accession number of SRP092778.

**Table 1:** Summary of the data for transcriptomes.

Sample	Insert size (bp)	Read length (bp)	Number of clean reads	Clean bases (bp)	Alignment togenome (%)	Alignment to CDS (%)
0 h	200	90_90	63,568,408	5,721,156,720	70.57	41.42
24 h	200	90_90	60,346,074	5,431,146,660	71.41	42.03
72 h	200	90_90	60,445,278	5,440,075,020	70.54	41.49

### Analysis of differential expression genes

**Fig 1:**
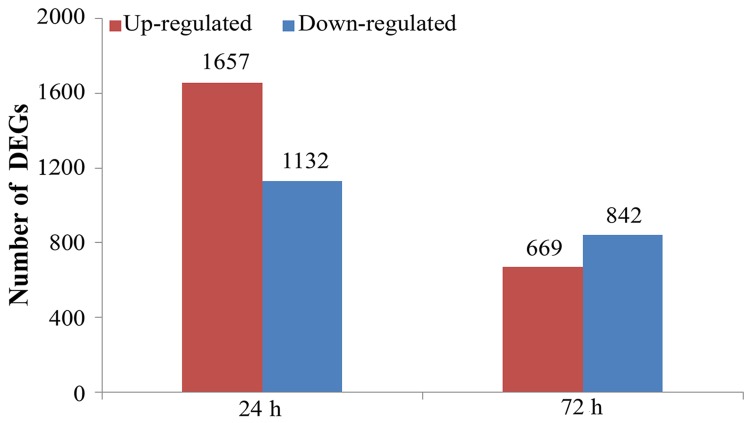
Statistic of differentially expressed genes (DEGs) at different time points after vaccination. The comparisons of gene expression difference between control (0 h) and each time point after vaccination (24 and 72 h) were performed based on FPKM values. The significant DEGs are defined as log_2_Ratio ≥ 1 and FDR ≤ 0.001.

The gene expression levels were calculated based on FPKM values, and gene expression difference was analyzed between each time point (24 and 72 h) after vaccination and control (0 h). At 24 h, 2,789 genes were differentially expressed (log_2_Ratio ≥ 1, FDR ≤ 0.001), including 1,132 up-regulated genes and 1,657 down-regulated genes (**Fig 1**). Meanwhile, a total of 1,511 differentially expressed genes were found at 72 h after vaccination, including 842 up-regulated genes and 669 down-regulated genes. All the differentially expressed genes were provided in **S2 Table**.

### Gene ontology and KEGG enrichment analysis of DEGs

**Fig 2:**
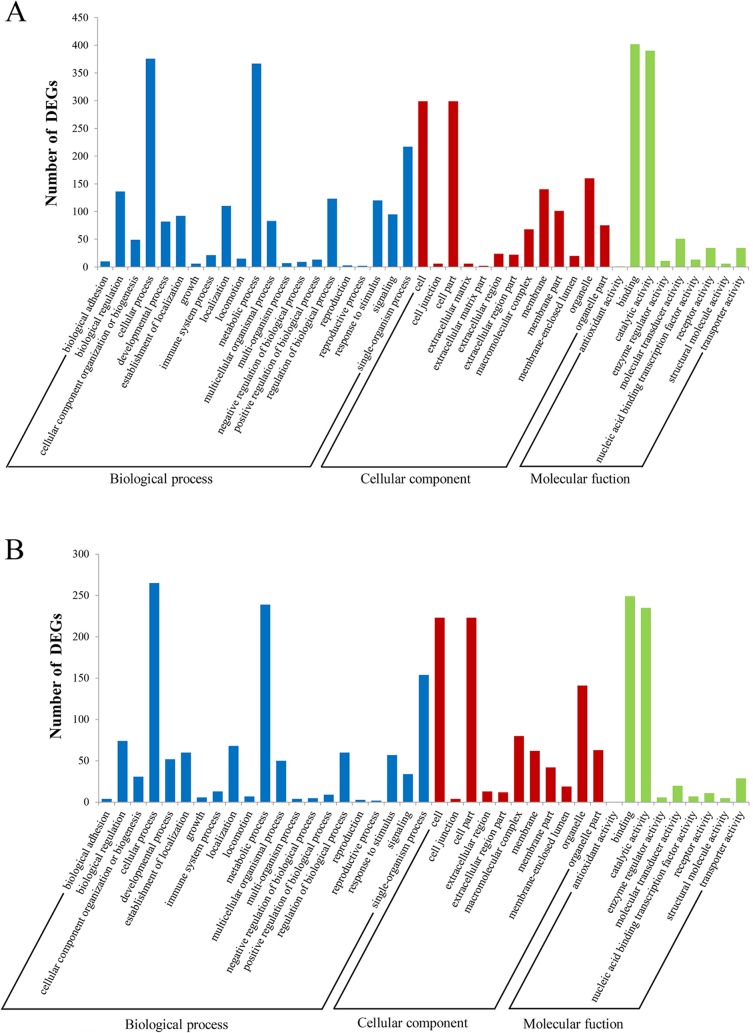
Enrichment of Gene Ontology classifications for the DEGs. The DEGs were annotated into three major functional categories: biological process, cellular component, and molecular function. The X-axis indicates the names of GO terms (2nd level) and the Y-axis indicates the number of DEGs. A, Gene Ontology classifications of DEGs at 24 h after vaccination; B, Gene Ontology classifications of DEGs at 72 h after vaccination.

Gene ontology functional enrichment analysis for DEGs at 24 h (or 72 h) showed that most of DEGs were classified into three major functional categories: ‘‘biological process”, ‘‘molecular function”, and ‘‘cellular component”, represented by 606 (or 389), 643 (or 390), and 420 (or 283) genes, respectively (**S3 Table**). The largest subcategories were “cellular processes” (299 or 223 genes), “catalytic activity” (390 or 235 genes), and “cell” (299 or 223 genes) at 24 h or 72 h in corresponding major functional categories. Moreover, two immune-relevant subcategories, “response to stimulus” (120 or 57 genes) and “immune system process” (21 or 13 genes) were also enriched (**Fig 2**).

KEGG analysis showed that 439 (or 279) DEGs were assigned to 216 (or 173) pathways at 24 (or 72 h) after vaccination, respectively (**S4 Table**). Immune-relevant KEGG pathways were listed in **Table 2,** including cytokine-cytokine receptor interaction (17 or 8 genes), T cell receptor signaling pathway (10 or 4 genes), NF-κB signaling pathway (14 or 4 genes), and antigen processing and presentation (8 or 4 genes) at 24 or 72 h. The metabolic pathway had the largest number of DEGs (66 or 39 genes, **S4 Table**), indicating that vaccination not only can activate immune-relevant pathways, but also may influence metabolic process.

**Table 2:** Immune-relevant KEGG pathways.

Pathway ID	Pathway name	Number of all * components	Number of DEGs (24 h)	Number of DEGs (72 h)
ko04020	Calcium signaling pathway	120	21	5
ko04514	Cell adhesion molecules (CAMs)	75	18	6
ko04060	Cytokine-cytokine receptor interaction	73	17	8
ko04142	Lysosome	69	16	4
ko04010	MAPK signaling pathway	110	16	6
ko04145	Phagosome	101	15	7
ko04144	Endocytosis	133	15	9
ko04064	NF-kappa B signaling pathway	64	14	4
ko04670	Leukocyte transendothelial migration	84	14	3
ko04062	Chemokine signaling pathway	80	11	4
ko04666	Fc gamma R-mediated phagocytosis	82	11	4
ko04660	T cell receptor signaling pathway	61	10	4
ko04650	Natural killer cell mediated cytotoxicity	50	9	2
ko04612	Antigen processing and presentation	25	8	4
ko04120	Ubiquitin mediated proteolysis	65	8	4

*: Be based on each relevant KEGG pathway.

### Annotation of immune-relevant genes

**Fig 3:**
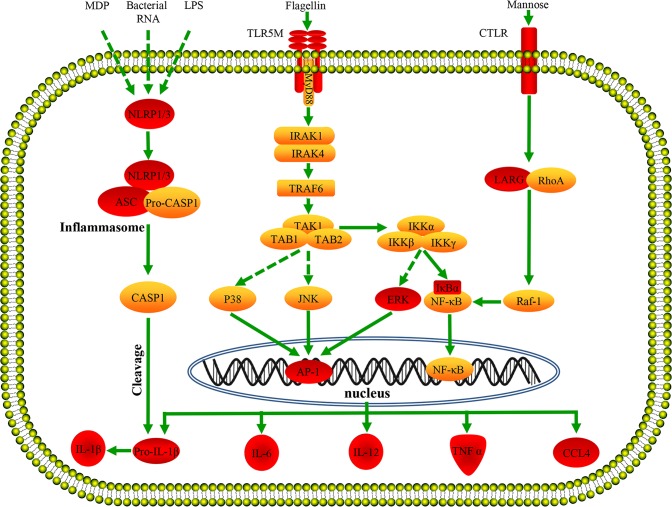
Pattern-recognition receptors and putative signaling pathways. The predicted pathway was constructed based on the references [43, 56]and the knowledge of mammalian species. Red background indicates significantly up-regulated expression, and yellow background indicates no significant differential expression. The arrows represent promotion, the solid lines indicate direct relationships between genes, and the dashed lines indicate that more than one step is involved in the process. After binding to flagellin, TLR5M triggers sequential recruitment of MyD88, members of IRAK (interleukin-1 receptor-associated kinase) family and TRAF6 that resulted in activation of nuclear factor NF- κB and AP-1, thus inducing production of inflammatory cytokines such as IL-1β, IL-6, IL-12, and TNF-α. After recognizing corresponding ligands (LPS, Muramyl Dipeptide, or bacterial RNA), NLRP1 or NLRP3 can bind with their adaptor proteins ASC and capase-1 to form the inflammasome complex, and the latter promotes cleavage and secretion of biologically active pro-inflammatory cytokine IL-1β.

To better understand the early immune response of large yellow croaker immunized with bacterial vaccine, the immune-relevant DEGs were collected and clustered (**Table 3**). Lots of important elements of innate immunity were significantly up-regulated at 24 or 72 h after vaccination, including complement components (C2, C3, C7, HF, BF, DF), coagulation system factors (F7, F8, THBS1), lectins (CLEC4E, COLEC12, CLEC17A, F-lectin 1, F-lectin 5, PTX), transferrins (TF, HP), antimicrobial proteins (BPI, lysG, Hep-1), pattern recognition receptors (TLR5M, MMR1, CTLR, PGRP-SC2, NLRP1, NLRP3, **Fig 3**), signal transducers (IκBα, ERK), transcriptional factors (RelB, AP-1), interleukins and interleukin receptors (IL-1β, IL-6, IL-10, IL-12, IL-27, IL-34, IL-1R, IL-12R, IL-22R), chemokines and chemokine receptors (CCL4, CCL19, CCL20, CCR1, CXCR3), apoptosis-related genes (CASP8, CASP10, BAX, BCL6), cathepsin (CTSL, CTSO), and other immune molecules (TNF-α, MMP-19).

**Table 3:** Immune-relevant DEGs found in the trivalent vaccine-induced transcriptome.

Accession number	Description	Gene name	log_2_ratio (24 h/0 h)	*P*-value	log_2_ratio (72 h/0 h)	*P*-value
**Innate defense molecules**						
Complement and coagulation cascades						
EH28_12969	Complement component 2	C2	-0.05	4.02E-01	1.03	3.83E-71
EH28_04156	Complement component 3	C3	1.64	0.00E+00	-0.07	0.00E+00
EH28_10105	Complement component 7	C7	3.38	4.54E-148	1.57	3.93E-18
EH28_10318	Complement factor B	BF	2.26	2.58E-08	1.67	2.39E-04
EH28_11857	Complement factor D	DF	1.19	0.00E+00	0.97	4.00E-308
EH28_04424	Complement factor H	HF	0.24	1.02E-76	1.25	1.22E-108
EH28_00866	Coagulation factor VII	F7	1.37	2.64E-01	2.24	3.10E-02
EH28_13616	Coagulation factor VIII	F8	2.73	0.00E+00	2.29	0.00E+00
EH28_04699	Thrombospondin-1	THBS1	3.28	0.00E+00	0.46	2.46E-04
Lectins						
EH28_17226	C-type lectin domain family 4 member E	CLEC4E	3.07	6.05E-169	1.98	4.42E-48
EH28_19458	C-type lectin domain family 17 member A	CLEC17A	0.69	3.39E-04	1.07	2.40E-09
EH28_17225	Collectin-12	COLEC12	6.56	5.78E-79	4.26	5.85E-14
EH28_00227	Fucolectin-1	F-lectin 1	-0.78	1.13E-06	1.67	2.17E-53
EH28_08704	Fucolectin-5	F-lectin 5	-0.82	2.00E-02	1.02	8.22E-05
EH28_17496	Pentraxin-related protein PTX3	PTX3	3.19	1.03E-09	1.9	4.52E-03
EH28_05767	Pentraxin-4	PTX4	4.50	4.21E-195	3.34	3.45E-70
Antibacterial proteins						
EH28_24821	Bactericidal permeability-increasing protein	BPI	0.49	1.33E-18	1.32	1.7E-167
EH28_14666	Haptoglobin	HP	4.36	0.00E+00	1.79	1.86E-35
EH28_06996	Hepcidin-1	Hep-1	3.34	1.10E-57	1.02	1.89E-03
EH28_15797	Lysozyme g	LysG	1.43	4.28E-31	1.34	3.20E-26
EH28_25262	Serotransferrin	TF	6.79	4.37E-92	4.9	8.76E-23
**Pattern recognition receptor signaling pathway**						
Pattern recognition receptors						
EH28_23426	C-type lectin receptor	CTLR	2.12	4.13E-26	0.82	1.30E-03
EH28_21627	Macrophage mannose receptor 1	MMR1	1.17	8.80E-112	1.26	1.02E-132
EH28_00392	NACHT, LRR and PYD domains containing protein 1	NLRP1	1.09	4.42E-02	0.47	5.78E-02
EH28_22777	NACHT, LRR and PYD domains containing protein 3	NLRP3	1.71	3.70E-37	1.42	3.70E-37
EH28_21234	Peptidoglycan-recognition protein SC2	PGRP-SC2	15.68	6.27E-33	13.46	1E-07
EH28_09444	Toll-like receptor 3	TLR3	-3.05	1.98E-81	-1.95	1.39E-48
EH28_18671	Toll-like receptor 5	TLR5M	3.71	1.07E-46	1.54	8.47E-08
Adaptor and signal transducers						
EH28_14997	Apoptosis-associated speck-like protein containing a CARD	ASC	1.10	5.17E-26	0.59	4.81E-07
EH28_25086	Mitogen-activated protein kinase 15	ERK	1.74	2.59E-08	0.62	1.05E-01
EH28_14297	NF-kappa-B inhibitor alpha	IκBα	1.90	0.00E+00	0.34	9.45E-11
EH28_20652	Putative ATP-dependent RNA helicase DHX58	LGP-2	-1.09	9.02E-89	-0.35	2.80E-13
EH28_12674	Rho guanine nucleotide exchange factor 12	LARG	1.61	7.84E-61	0.51	2.00E-05
EH28_06883	Transcription factor AP-1	Ap-1	2.13	7.56E-89	0.12	4.53E-01
**Cytokines and Cytokine receptors**						
Cytokines						
EH28_13993	C-C motif chemokine 4	CCL4	2.46	2.86E-87	2.52	1.91E-92
EH28_23981	C-C motif chemokine 19	CCL19	2.64	0.00E+00	0.77	5.43E-54
EH28_25343	C-C motif chemokine 20	CCL20	3.71	5.25E-09	1.33	1.95E-01
EH28_21923	Interleukin-1 beta	IL-1β	5.16	5.92E-91	3.3	3.22E-19
EH28_25178	Interleukin-6	IL-6	5.19	1.09E-37	2.41	5.79E-04
EH28_18941	Interleukin-10	IL-10	4.14	1.73E-14	3.02	5.09E-24
EH28_23359	Interleukin-12 subunit beta	IL-12	3.64	1.55E-28	0.43	4.72E-01
EH28_14432	Interleukin-27 subunit beta	IL-27	1.80	1.67E-15	1.19	2.61E-06
EH28_03825	Interleukin-34	IL-34	1.03	1.39E-06	1.06	6.59E-07
EH28_07798	Interferon gamma	IFN-γ	1.65	2.82E-04	1.93	8.9E-06
EH28_05037	Tumor necrosis factor alpha	TNF-α	2.07	5.97E-04	0.50	5.39E-01
Cytokine receptors						
EH28_03117	C-X-C chemokine receptor type 1	CXCR1	1.92	1.33E-226	0.64	2.45E-17
EH28_21430	C-X-C chemokine receptor type 3	CXCR3	1.19	6.26E-47	0.55	3.52E-09
EH28_18535	Interleukin-1 receptor type 2	IL-1R	7.32	0.00E+00	4.62	3.18E-99
EH28_07447	Interleukin-12 receptor subunit beta-2	IL-12R	1.26	8.92E-122	0.27	4.40E-05
EH28_04343	Interleukin-22 receptor subunit alpha-2	IL-22R	1.65	8.52E-05	0.19	7.50E-01
**Apoptosis-related genes**						
EH28_19418	Apoptosis facilitator Bcl-2-like protein 14	BCL2L14	1.97	1.60E-34	1.55	4.60E-19
EH28_07897	Apoptosis regulator BAX	BAX	1.04	5.90E-05	1.22	1.05E-06
EH28_10711	B-cell lymphoma 6 protein	BCL6	1.27	1.39E-31	0.45	3.09E-04
EH28_18771	Caspase-8	CASP8	1.13	2.03E-24	0.53	2.58E-05
EH28_18769	Caspase-10	CASP10	1.07	2.91E-22	1.02	5.27E-20
**Antigen processing and presentation**						
EH28_19874	Antigen peptide transporter 1	TAP1	2.38	0.00E-00	1.21	5.21E-80
EH28_06369	Antigen peptide transporter 2	TAP2	1.28	1.66E-71	0.90	1.21E-31
EH28_09011	Calreticulin	CLAR	1.53	1.60E-01	1.47	2.12E-178
EH28_06817	Cathepsin L	CTSL	1.23	1.24E-168	1.12	1.03E-131
EH28_11008	Cathepsin O	CTSO	1.10	3.35E-07	0.67	4.33E-03
EH28_11216	Endoplasmic reticulum aminopeptidase 1	ERAP1	1.32	6.11E-86	0.86	6.17E-32
EH28_24090	Endoplasmic reticulum aminopeptidase 2	ERAP2	1.76	1.99E-92	0.90	3.73E-19
EH28_14158	Heat shock protein 70KDa-4	HSP70	1.35	1.61E-47	0.83	4.94E-16
EH28_21169	HLA class II histocompatibility antigen gamma chain	MHC- II	-0.19	1.42E-36	-0.71	0.00E+00
EH28_17090	Lysosome-associated membrane glycoprotein 1	LAMP-1	2.08	0.00E+00	1.13	8.36E-100
EH28_25045	Lysosomal thioesterase PPT2-A	PPT2	1.53	1.00E-05	0.74	7.00E-02
EH28_00289	Major histocompatibility complex, class I	MHC-I	0.75	2.09E-299	0.32	3.49E-125
EH28_18380	Proteasome activator subunit 1	PA28α	1.11	9.93E-113	1.12	1.05E-114
EH28_02969	Proteasome activator complex subunit 2	PA28β	1.55	2.06E-202	1.2	5.77E-106
EH28_21964	Proteasome subunit alpha type-1	PSMA1	1.24	6.62E-118	1.18	9.09E-105
EH28_21059	Proteasome subunit alpha type-4	PSMA4	1.25	1.15E-66	1.37	1.01E-82
EH28_10635	Proteasome subunit beta type-2	PSMB2	1.28	1.22E-69	1.37	2.58E-82
EH28_20274	Proteasome subunit beta type-7	PSMB7	1.38	1.19E-82	0.95	7.43E-34
EH28_19428	Tapasin	TAPBP	1.06	1.35E-39	0.38	2.62E-09
**TCR signaling pathway**						
EH28_05511	CD3 gamma chain	CD3γ	-1.20	2.01E-12	-0.41	3.65E-03
EH28_19424	Protein kinase C theta type	PKCθ	-0.92	6.35E-10	-0.34	1.01E-02
EH28_04099	Pyruvate dehydrogenase kinase isozyme 2	PDK	-1.52	2.24E-118	-1.33	5.55E-97
EH28_02274	T-cell receptor alpha chain V region	TCRα	-1.28	6.49E-05	-0.66	2.06E-02
EH28_23685	T-cell receptor beta chain T17T-22	TCRβ	-1.18	1.13E-23	0.02	9.01E-01
EH28_17224	T-cell surface glycoprotein CD4	CD4	-1.85	2.66E-42	-0.31	2.35E-03
EH28_09327	T-cell surface glycoprotein CD8 beta chain	CD8β	-1.70	1.38E-02	-1.00	9.95E-02
EH28_16421	Tyrosine-protein kinase Fyn	FYN	-1.07	9.37E-25	-0.71	2.86E-13
EH28_09736	Tyrosine-protein kinase ZAP-70	ZAP-70	-1.44	2.35E-09	0.18	3.62E-01
EH28_23772	1-phosphatidylinositol-4,5-bisphosphate phosphodiesterase gamma-1	PLC-y1	-1.33	8.40E-40	-0.62	2.70E-12

**Fig 4:**
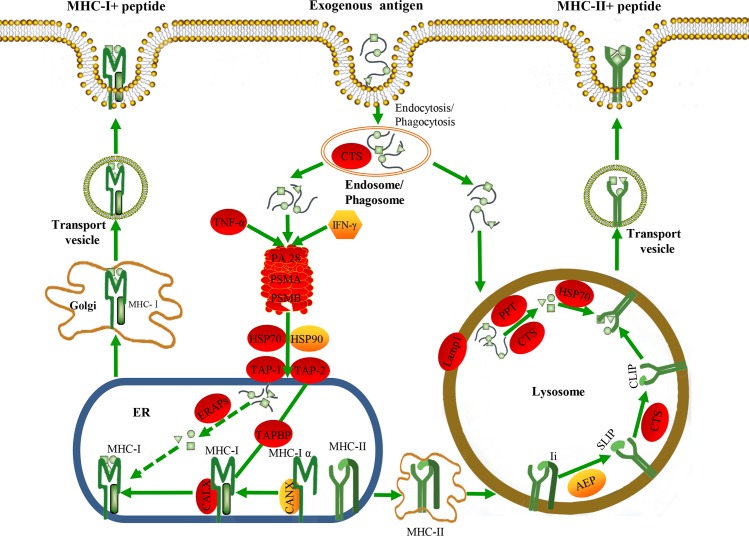
Putative antigen processing and presentation pathway. The predicted antigen processing and presentation pathway of large yellow croaker was constructed based on the knowledge of mammalian species. Red background indicates significantly up-regulated expression, and yellow background indicates no significant differential expression. The arrows represent promotion, the solid lines indicate direct relationships between genes, and the dashed lines indicate that more than one step is involved in the process. Bacterial antigens are internalized into host cells via phagosomes or endosomes. Specifically, the antigenic peptides are degraded in the cytoplasm by proteasome, and then transported to the endoplasmic reticulum by TAP and loaded onto MHC-I molecules with the help of ERAP, TAP, TAPBP, and CLAR. Antigenic peptides can also be captured and degraded in endosome and lysosome with the help of CTS, HSP70, and PPT2, and then loaded onto MHC-II molecules.

Additionally, many adaptive immune-relevant genes, such as proteasome molecules (PA28, PSMA, PSMB), antigen processing-related genes (CLAR, TAP, TAPBP, HSP70, **Fig 4**), and regulatory factors (IFN-γ, IL-10, TNF-α) were up-regulated after vaccination, whereas T cell receptor (TCR) signaling molecules (CD3γ, CD4, CD8, TCRα, TCRβ, FYN, ZAP-70, PLC-γ1, **Table 3**), and some immunoglobulins (Ig heavy chain V region, Ig heavy chain V-III region CAM, Ig heavy chain V-III region HIL, Ig kappa chain V-IV region JI) were down-regulated (**S2 Table**).

### Verification of the expression changes of partial immune-relevant genes

**Fig 5:**
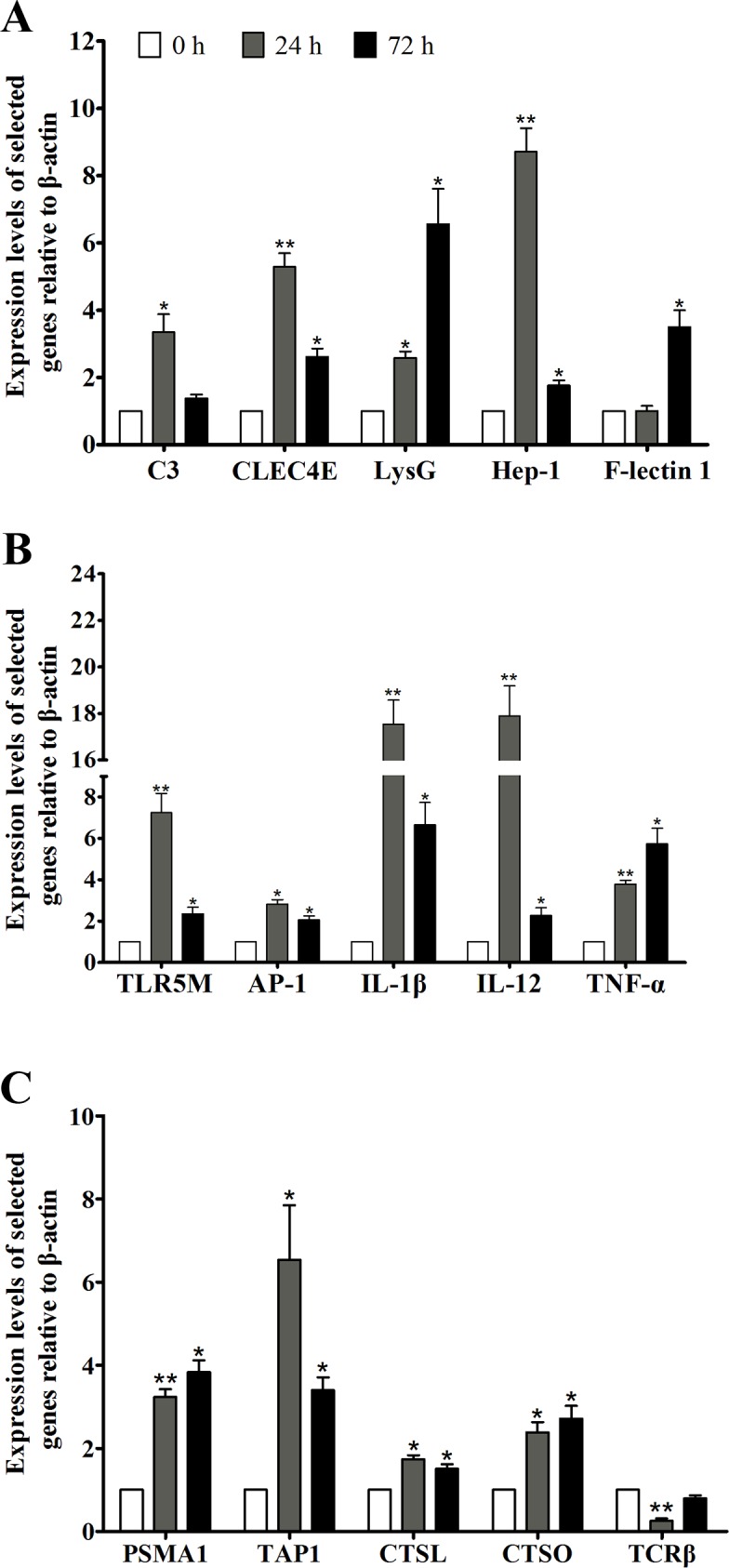
Real-time PCR analysis of selected DEGs. Real-time PCR was used to validate DEGs associated with innate defense (A), ligand recognition and signal transduction (B), antigen processing and presetation and TCR signaling pathway (C). The expression levels of target gene were expressed relative to those of β-actin in each sample using the 2^-ΔΔCT^ method. Two-tailed Student’s *t* test was used for the significance test analysis, ^*^P < 0.05, ^**^P < 0.01.

Partial DEGs were validated by quantitative real-time PCR. The mRNA transcripts of C3, CLEC4E, F-lectin 1, LysG, Hep-1, TLR5M, AP-1, IL-1β, IL-12, TNF-α, TAP1, PSMA1, CTSL, and CTSO were observably increased at 24 or 72 h after vaccination, while the expression levels of TCRβ were down-regulated (**Fig 5**). Fold changes of those genes from quantitative real-time PCR were well corresponding to the results of RNA-seq expression analysis, supporting the reliability of the transcriptome data.

## Discussion

To date RNA-seq based transcriptome profiling has been widely used in fish for identifying host determinants of response to bacterial infection [22, 23, 31–33]. In previous study, we have demonstrated that *A*. *hydrophila* infection could activate Toll-like receptor, JAK-STAT, and MAPK pathways, while inhibit TCR signaling pathway at the early period in large yellow croaker [34]. In this study, we analyzed the spleen transcriptome profiles of large yellow croaker after vaccination and found abundant DEGs were involved in innate defense, ligand recognition, antigen processing, and TCR signaling processes.

Innate immune response is considered as the first line of host defense in opposing pathogenic infection in fish. The components of the innate defense are evolutionarily conserved in organisms, including antimicrobial peptides, lysozymes, complement, lectins, and transferrins [35]. In this study, the expression of several innate defense molecules was increased obviously after vaccination (**Table 3)**. Their up-regulations were further confirmed by real-time PCR, which were well corresponding to the results from transcriptome analysis (**Fig 5A).** The similar findings were also found in the studies on Atlantic salmon [11]and Atlantic cod [13], indicating that bacterial vaccine immunization could significantly increase the expression of innate immunity molecules in fish. The activation of complement system usually initiates a cascade of biochemical reactions, resulting in antigen elimination via cell membrane lysis [36]. C3 and C7 of large yellow croaker have been proved to participate in the response to bacterial challenge [37, 38]. Lectins possess binding activity towards carbohydrate residues located on bacterial surfaces and aid neutralization of pathogens [39]. The transferrins, lysozymes, and antibacterial peptides function as the growth inhibitors of bacteria against the infection of bacterial pathogens [15, 40, 41]. These data suggest that immunization with trivalent vaccine intensely enhances the innate defense of large yellow croaker.

The activation of innate immune system against invading microbes mainly relies on the recognition of microbial components by pattern-recognition receptors (PRRs) [42]. Several PRRs, including TLR5M, NLRP1, NLRP3, MMR1, PGRP-SC2, and CTLR were up-regulated significantly after vaccination (**Table 3** and **Fig 5B**). TLR5M is known to recognize bacterial flagellin, signal through MyD88 and TRAF6, trigger the activation of MAPK and NF-κB, and induce inflammatory factors production [43]. Due to *V*. *alginolyticus*, *V*. *parahaemolyticus* and *A*. *hydrophila* are all flagellated bacteria, TLR5M was up-regulated by 7.8-fold at 24 h after vaccination with trivalent bacterial vaccine. Eventually, its downstream signal transducers (TRAF6, IκBα, ERK), transcriptional factors (RelB, AP-1), and effectors (TNF-α, IL-6, IL-12, CCL4) were also up-regulated, indicating TLR5M mediated signaling pathway was activated by trivalent vaccine (**Fig 3)**. Other signal transducers, such as IRAK1/4, TAK1, and IKKα/β/γ were changed slightly after vaccination, perhaps due to these genes play their roles through the changes in phosphorylation levels [44]. NLRP1 or NLRP3 can bind with their adaptor proteins ASC and capase-1 to form the inflammasome complex after recognizing corresponding ligands (LPS, Muramyl Dipeptide, or bacterial RNA), and then promote cleavage and secretion of biologically active pro-inflammatory cytokine IL-1β [45–47]. In this study, NLRP1, NLRP3, ASC, and IL-1β were significantly induced by vaccination (**Fig 3**), which may result in increase of the biologically active IL-1β to recruit inflammatory cells for eliminating pathogens. These results demonstrate that multiple innate immune processes were activated at the early stage of vaccination in large yellow croaker.

Spleen is a major secondary lymphoid organ in the fish, containing a large number of B cells, T cells, neutrophils, and macrophages. The Neutrophils and macrophages as antigen-presenting cells, possess heterogeneous intracellular pathways responsible for generating complexes of MHC class I and class II molecules with peptide antigens, for presentation to T cells [48–51]. Bacterial antigens are internalized into host cells via phagosomes or endosomes. Specifically, the antigenic peptides are degraded in the cytoplasm by proteasome, and then transported to the endoplasmic reticulum by TAP and loaded onto MHC-I molecules with the help of ERAP, TAP, TAPBP, and CLAR [52]. Antigenic peptides also can be captured and degraded in endosome and lysosome with the help of CTS, HSP70, and PPT2, and then loaded onto MHC-II molecules [53]. In our study, CTS, PPT2, CLAR, TAP, HSP70, and TAPBP were significantly up-regulated after vaccination (**Fig 4**), while slightly changes were found in the MHC-I and MHC-II expression levels (**Table 3**). These results suggest that antigen processing may be activated at the early stage of vaccination, but the antigen presentation was in the process of being started.

Moreover, the expression levels of several genes involved in TCR signaling pathway, including TCRα, TCRβ, related CD molecules (CD3γ, CD4, and CD8β), and downstream signaling molecules (FYN, ZAP-70, PLC-γ1, PDK, and PKCθ), were down-regulated by trivalent bacterial vaccine (**Table 3**), suggesting that the TCR signaling pathway may be suppressed at the early stage of vaccination. The similar results were also found in other teleost fish [22, 34, 54, 55]. In zebrafish, expression of TCR pathway-related genes was found to decrease at 24 h following immunization with attenuated *Edwardsiella tarda* vaccine [54]. In half-smooth tongue sole (*Cynoglossus semilaevis*), TCR pathway was suppressed at 20 h after *Vibrio anguillarum* infection [55]. Expression levels of TCRα, β, and δ in tilapia (*Oreochromis niloticus*) were down-regulated by *Streptococcus iniae* at 24 h post-infection [22]. In our previous study, TCR pathway was also suppressed at 24 h after *Aeromonas hydrophila* infection in large yellow croaker [34]. However, the mechanism by which TCR pathway was suppressed at the early period of immunization with bacterial vaccine or bacterial infection remains unknown.

In conclusion, transcriptome analysis was utilized to investigate the early immune response of large yellow croaker to bacterial vaccine immunization. The most of strongly up-regulated genes are innate defense molecules, bacterial ligand—depending PRRs, inflammatory factors, and antigen processing related molecules, indicating that multiple innate immune processes and acquired immune initiation were activated at the early stage of vaccination in large yellow croaker. However, TCR signaling pathway was suppressed after vaccination. The findings observed in this study are similar to immune responses of large yellow croaker infected by live *A*. *hydrophila*, implying that inactivated vaccine has the similar effects on induction of host defense by bacterial infection. These results comprehensively reveal the early immune response in the large yellow croaker induced by trivalent vaccine and provide valuable information for developing a highly immunogenic vaccine in teleosts.

## Supporting Information

S1 Fig Agarose gel electrophoresis analysis of the specificity of primers. (PDF)

S1 Table Primer sequences for real-time PCR. (DOC)

S2 Table Details of all differentially expressed genes identified in the spleen transcriptomes of the large yellow croaker after vaccination. (XLS)

S3 Table Gene Ontology classifications of all DEGs at 24 and 72 h after vaccination. (XLS)

S4 Table KEGG mapping of all DEGs at 24 and 72 h after vaccination. (XLS)
