# Supplementary figures and images for: Transcriptome Analysis Reveals Comprehensive Insights into the Early Immune Response of Large Yellow Croaker (Larimichthys crocea) Induced by Trivalent Bacterial Vaccine
Source: PLoS One. 2017 Jan 30;12(1):e0170958. doi: 10.1371/journal.pone.0170958 (PMC5279777; doi:10.1371/journal.pone.0170958)

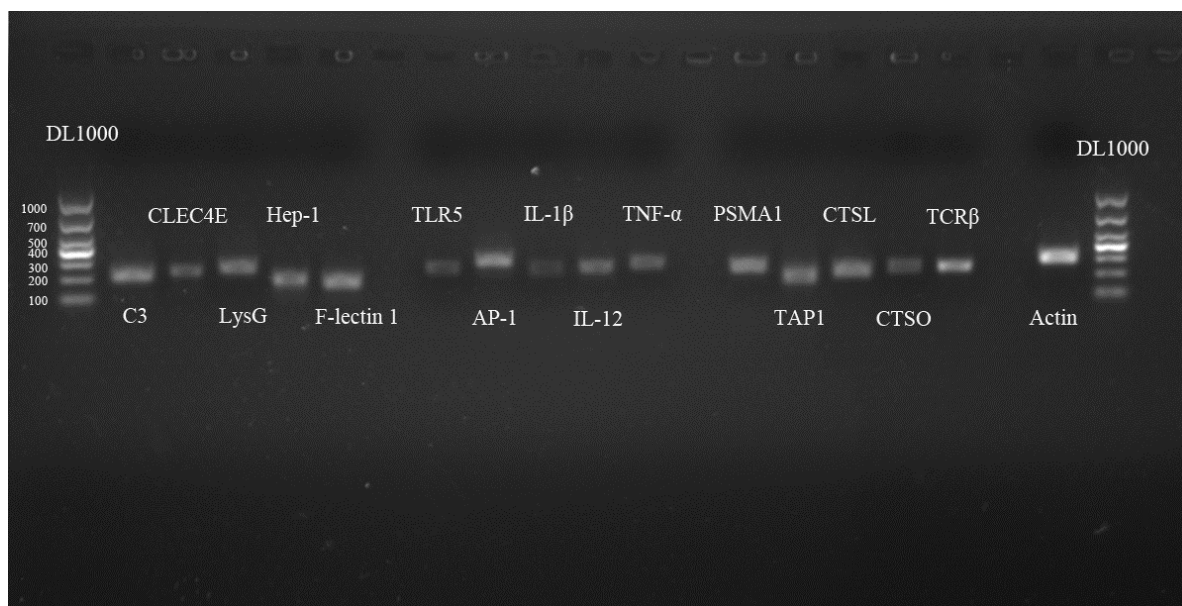

Supplement: S1 Fig — (PDF) [file pone.0170958.s001.pdf]
