# Supplementary material for: Transcriptome Analysis Reveals Comprehensive Insights into the Early Immune Response of Large Yellow Croaker (Larimichthys crocea) Induced by Trivalent Bacterial Vaccine
Source: PLoS One. 2017 Jan 30;12(1):e0170958. doi: 10.1371/journal.pone.0170958 (PMC5279777; doi:10.1371/journal.pone.0170958)
Supplement: S1 Table — (DOC) [file pone.0170958.s002.doc]

**Table S1. Primer sequences for real-time PCR**

| **Gene name** | **Forward Primer (5’-3’)** | **Reverse Primer (5’-3’)** |
| --- | --- | --- |
| β-actin | GACCTGACAGACTACCTCATG | AGTTGAAGGTGGTCTCGTGGA |
| C3 | CCCATTGAGGTCAAAGCAGC | TGGAAGGTGTGTTTGGGACC |
| CLEC4E | AAGGGAACAGGCCCATTCTG | TAACGTCTGCGCATTGAGGT |
| LysG | ACTACCGGTGCATCATTGGA | TTGTCCTCTCGCTGGGCTAT |
| Hep-1 | ACACTCGTGCTCGCCTTTAT | GTAGCGGCACAAGGAGATGT |
| F-lectin1 | CCTTCATGGCAACCCAACCT | CTCACCAGTTTTGGCACGGT |
| TLR5 | AGCTCCAACGCACTACAGTC | AGCCATTCAGGTTGTGATTGC |
| AP-1 | CAGTCCAGCAACGGACTCAT | TCAAGTCCTCGTAAACCGGC |
| IL-1β | AGACAGCGGATTACCAGTGC | TCCCCATCCCTATGGCAAGA |
| Il-12 | TGAAACCTGGCAAGGTGAGG | TCGGATACAGACGGCTTTGG |
| TNF-α | GGACGATTCTTCGTTTACAG | GTTTGTCACCTCTGTTCAGG |
| PSMA1 | GACGGACAGCTACTTTCGCA | ACAGAGCAGTCTGGCATCAG |
| TAP-1 | AGGATGACAAAAGCGGCATC | AATGCATCAGGCTCCTCTTCG |
| CTSL | CAAGGAGCATGCACTTATGAAGG | TTCACGATCCAGTATTTCTTACCG |
| CTSO | CAAGCGTGTGGGAGTTGTTG | GCAGATTCCAGTCTTGGCCT |
| TCRβ | TTCAAGGCGTCTGTCGAAGC | GGTCTGGGTCGAGAACAGTC |
